# “Am I Masculine?” A metasynthesis of qualitative studies on traditional masculinity on infertility

**DOI:** 10.12688/f1000research.131599.1

**Published:** 2023-03-09

**Authors:** Cennikon Pakpahan, Raditya Ibrahim, William William, Patricia S Kandar, Darmadi Darmadi, A. ST. Aldilah Khaerana, Supardi Supardi

**Affiliations:** 1 Andrology Study Program, Faculty of Medicine, Universitas Airlangga, Surabaya, East Java, 60132, Indonesia; 2 Department of Biomedical Science, Faculty of Medicine, Universitas Airlangga, Surabaya, East Java, 60132, Indonesia; 3 Department of Medical Biology, School of Medicine and Health Sciences, Universitas Katolik Indonesia Atma Jaya, South Jakarta, Special Capital Region of Jakarta, 14440, Indonesia; 4 Department of Internal Medicine, Faculty of Medicine, Universitas Sumatera Utara, Medan, North Sumatra, 20155, Indonesia; 5 English Department, Faculty of Culture Sciences, Universitas Hasanuddin, Makassar, South Sulawesi, 90245, Indonesia

**Keywords:** feminism, gender equality, infertility, masculinity, psychological well-being, metasynthesis, qualitative

## Abstract

**Background**: The rate of infertility is increasing day by day. According to studies conducted worldwide, 30 million men are diagnosed with infertility. Cases of infertility are often associated with a failure to become male in society. Procreation and gender roles are often closely linked so that infertile men are often considered the second sex. Sometimes, this condition makes men question their masculinity.

**Methods**: We performed a systematic review and metasynthesis with the Preferred Reporting Items for Systematic Reviews and Meta-Analyses guideline procedure on qualitative studies on ten databases exploring the experience of infertile men and their association with masculinity.

**Results**: Twenty-four studies matched our question, and there are two major themes with eight subthemes that were obtained from the results of the metasynthesis of these studies. The impact of this gender issue is huge on men’s health and their social interactions. As a result, gender issues provide a space for debate and a burden on men. Sometimes, men develop mental health problems. The topic of masculinity and infertility is at odds with feminism and is susceptible to the societal stigma that results from the hegemonic conception of masculinity. Interestingly, the men must accept reality and follow the treatment process for infertility, although it affects their psychological well-being.

**Conclusions**: These findings provide insight for physicians, as treating infertility requires a multidisciplinary team that does not only address procreation issues. Social issues related to gender roles often bring patients into harmful and dangerous conditions. To address the gender issue in men globally in several dimensions, however, a large study in various populations is still required.

## 1. Introduction

Creating a family is one of the reasons for people to get married. Newlywed couples are often expected to have children. But for those couples who take years to have children or even do not get the chance to, it can become an issue. Infertility for some couples can mean they have to make extra effort to get descendants. In some communities, it is undeniable that infertility has a significant impact on increasing number of divorce cases, economic difficulties, and even cases of losing the right to a funeral ( Greil *et al.*, 2010; Kumar & Singh, 2015). Infertility can be defined as the inability of a couple to become pregnant after 12 months of regular sexual intercourse without the use of contraception or protection ( Zegers-Hochschild *et al.*, 2009). Infertility affects 8%–12% of couples globally, and 20%–30% of cases are due to an issue with the male ( Agarwal *et al.*, 2015). It has been reported that there is a decline in the quality of male semen in several countries around the world ( Dissanayake *et al.*, 2019). Levine *et al.* reported that in the last 40 years, 1 in 20 men faced infertility problems ( Levine *et al.*, 2017). It is also confirmed by Agarwal *et al.* who said that at least 30 million men worldwide are confirmed infertile. The global rate of male infertility cases range from 2.5% to 12%. The highest numbers were found in Africa and Eastern Europe. This number indicates that infertility can occur anywhere ( Agarwal *et al.*, 2021).

Men are reported to struggle when realizing that they are infertile. Feelings of vulnerability, isolation, failure, and futility are commonly found ( Ravitsky & Kimmins, 2019). In some communities, it is undeniable that infertility has a significant impact on the increasing number of divorce cases, economic difficulties, and even cases of losing the right to a funeral ( Greil *et al.*, 2010; Kumar & Singh, 2015).

There is an intriguing relationship between the incidence of male infertility and its impact on men from a psychological and social perspective. These are inextricably linked to the persisting stigma surrounding infertility, which can lower a man’s self-esteem. This concept often means that infertile men are viewed as the “second sex” or inferior masculinity. Infertile males are considered deviants based on hegemonic masculinity norms; infertility drives men to reconstruct and renegotiate their own masculine identity. This is influenced by the impact of hegemonic masculinity that is prevalent in parts of the world with different races and cultures ( Burton, 2014).

### 1.1 Understanding masculinity

The public often uses the term masculinity to define men as masculine while women are feminine. Masculinity has been viewed as strength, power, and aggressiveness and is formed in opposition to fear, anxiety, or weakness ( Burton, 2014; Rotundi & Pitti, 2020). Men who have a muscular body and fulfill specific “criteria” are frequently considered masculine. Being fertile is also considered a part of masculinity ( Burton, 2014; Rivera & Scholar, 2020) This assumption often implies that masculinity is brought from birth.

Many experts argue that masculinity is formed by the interaction of various factors. For example, historical and sociocultural factors, and geographic location—and is not innate from birth ( Rotundi & Pitti, 2020). According to the social constructivist theory of masculinity, gender is achieved through and by people and their environments. Nowadays, the ostensible boundary between gender (one’s role in society) and sex (physiology reproductive function) is vanishing. Gender is something we perform in social interactions, not an innate part of us ( Moynihan, 1998). Society tried to formulate the idea of masculinity based on the results of these interactions. Therefore, we have to be careful because it is possible that this viewpoint can lead to “hegemonic masculinity,” a condition where every man will race to be recognized as a “real” man ( Connell & Messerschmidt, 2005).

Currently, it is undeniable that there has been a shift in society, especially in the population of young men. This community is more courageous in expressing their tenet socially coded, traditionally, as feminine ( Anderson, 2018). More men are aware that masculinity is something they do and perform rather than what they are ( Waling, 2019). The traditional masculinity concept argues that a man must be strong, not show many emotions, and must be independent and dominant. The virility means that a man can make a woman pregnant and function sexually. On the contrary, a male that does not fit these characteristics is not considered a real male. This act of overly understanding the traditional masculinity concept results in masculinity as toxic ( Gannon *et al.*, 2004; Rivera & Scholar, 2020).

Traditional masculinity can limit personal growth for a man and can cause distress to those who do not meet those criteria. This can lead to depression, substance use, and even suicide ( Gannon *et al.*, 2004; Kupers, 2005). Traditional masculinity can also make a man feel shame for seeking help when in distress, mentally or physically, because of the image of being less masculine. The American Psychological Association (APA) argues that men who try to adapt to the traditional masculinity concept are more prone to have mental or physical problems ( Gannon *et al.*, 2004; Kupers, 2005). This underlies the APA to issue Guidelines for Psychological Practice with Boys and Men in August 2018. Boys raised in a traditional masculine household have a mindset that they must adopt this behavior to be accepted in society. Traditional masculinity can be found in everyday life, such as bullying, fighting, breaking the law, sexual assault, domestic violence, criminal acts, and drug use.

### 1.2 Masculinity and infertility

Formerly, there are minimal social studies that explore the experience of infertile men accepting their roles from a gender perspective ( Hanna & Gough, 2015). This is because reproductive biological function is identical to “feminine area that demands female commitment and labour,” in part due to our gendered culture, yet “male procreative contributions and reproductive masculinity” are represented as unproblematic, with males being deemed fertile throughout their lifespan ( Hanna & Gough, 2020).

There is a perception that success and masculinity go hand in hand, especially in Western myths. Having an illness related to sex roles, causes men to suppress their emotions ( Moynihan, 1998). Social construction demands men to be fertile. This condition makes it very difficult for men to accept pain or express fears and weaknesses, most notably when they are dealing with infertility cases.

Infertility as a medical issue and the significance of patriarchal authority are closely related. Androcentric medical knowledge constructs, legitimizes, and maintains gender norms, reflecting Western social and cultural notions of gender, in which rigid definitions of gender normativity are constructed, and any behavior or trait that exists outside these norms is considered deviant ( Conrad & Barker, 2010).

Infertile men lack these characteristics and are hence perceived as inadequate. As a result, infertility is a sign of a lack of masculinity because it does not fit within these ideals, labeling the person as less masculine ( Dudgeon & Inhorn, 2003). Infertility poses a danger to masculinity because of the faulty belief that anything that exists outside the norms of masculinity must be attributed to femaleness, and thus males who break from these norms go closer to embodying female characteristics. In this sense, infertile males are connected with characteristics associated with femininity, such as being weaker and in need of assistance ( Gannon *et al.*, 2004). “Men’s sexuality is sanctioned and encouraged, whereas women’s sexuality may be rigorously controlled, limited, and condemned,” according to a system of binary oppositions maintained by hegemonic masculine ideologies ( Dudgeon & Inhorn, 2003).

Finally, male infertility has lately been found to be perceived by men as a failure of manhood, a process fraught with stigma, silence, and suppression, as well as having a devastating and alienating effect on their life ( Hanna & Gough, 2020).

In the following section, we try to investigate gender-based studies conducted on infertile men, providing an overview of the association between masculinity and male infertility. This study aims to explore the phenomenon of masculinity in cases of infertility

## 2. Methods

### 2.1 Criteria for inclusion and exclusion

The researchers/writers included the following studies: 1) qualitative studies using in-depth direct conversation or an online survey with the sample, 2) studies in English, 3) studies that contain information about gender issues, and 4) a study that was in a male infertility setting. Meanwhile, the researchers/writers excluded 1) literature review studies or other reviews, 2) quantitative studies, 3) studies that do not provide complete data such as information about gender issues, 4) Masculinity studies with qualitative methods but not on cases of infertility, 5) studies that are not in English, and 6) studies that are not available in full text.

### 2.2 Database searching

**Figure 1.**
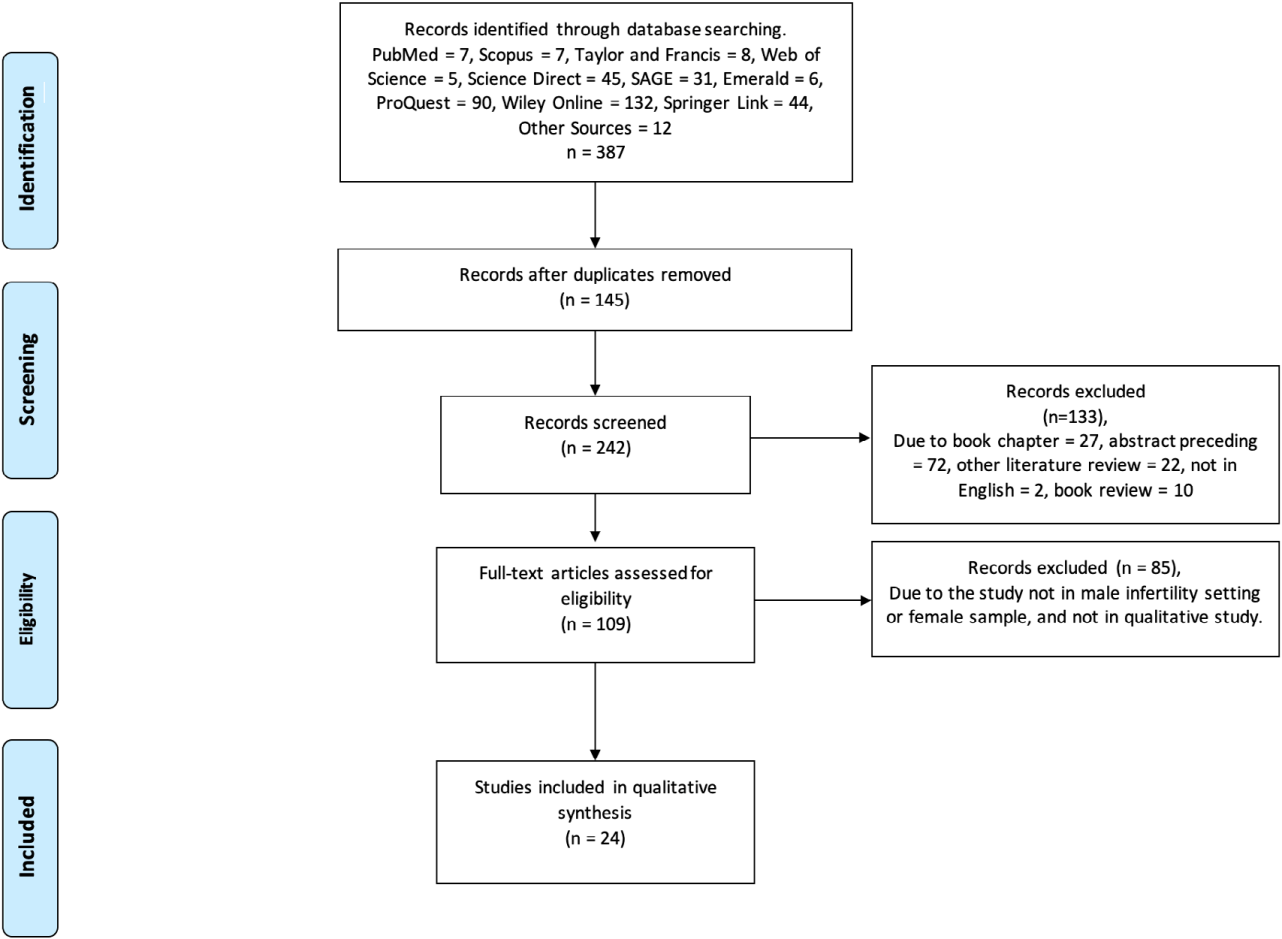
PRISMA flow chart for study findings.

To produce an adequate report on this systematic review, the researchers collected the data according to the Preferred Reporting Items for Systematic Reviews and Meta-Analyses (PRISMA) guidelines ( Figure 1) ( Page *et al.*, 2021). This study was registered on PROSPERO with registration number CRD42022327318.

In this systematic review, a study search was conducted in ten databases, such as PubMed, SAGE, Scopus, Taylor & Francis, Web of Science, ScienceDirect, Emerald, ProQuest, Wiley Online, and Springer Link. Other studies taken from sources (Google Scholar, researchsquare, medRix) that had not been mentioned earlier were also gathered. To search for studies involving the specific gender theme of “masculinity” in male infertility, the researchers used “gender” OR “masculinity” AND “male infertility” as keywords. The study was presented qualitatively with regard to the information on gender phenomenon not according to the infertility problems they faced.

### 2.3 Selection of the studies

Two authors (CP and RI) conducted a separate study electronically using a previously approved keyword then used Mendeley to assess all the studies. The researchers (CI, RI, WW, and PSK) collected and retrieved the search and then filtered out the studies that fit the required theme. Any confusion that evolved during the search was discussed with the other authors (DD and SS).

### 2.4 Data collection

After the two authors (CP and RI) separated the studies according to Mendeley. CP, RI, WW, and PSK collected data together from studies that met the criteria. The researcher discussed the results/findings and then wrote the report. The year, author, location or population, sample size, title, and investigation results, including gender theme, were obtained from each study. If the required information was missed, we attempted to contact the article’s corresponding author. We conducted a metasynthesis after collecting the data completely.

### 2.5 Quality and risk bias assessment

The critical appraisal skills program (CASP) was used to evaluate the quality of the studies that were included. This scale contained ten questions that evaluated the feasibility or quality of the included qualitative study. Each question would hold choices of “yes,” “no,” or “can’t tell” ( CASP, 2018). The assessment using this scale was only to evaluate whether the methodology of this study/article is appropriate or not. The assessment of this scale was carried out by two authors independently and then discussed the conclusion.

### 2.6 Theme synthesis

The researchers of this study conducted a review synthesis of the extracted journals. The synthesis consisted of two stages. The first stage was identifying the characteristics of the collected studies. We drew demographic data, research methods, and the number of samples used and then concluded the research results related to gender issues. The second stage was an in-depth review that was conducted using inductive phenomenology. At this stage, we explored descriptive information on how the experiences and interactions felt by the subjects comprehensively on whatever gender issue phenomenon they thought were related to the experience of daily awareness as infertile patients. The extracted data included perceptions (what the respondents hear and see), beliefs, memories, and feelings that are experienced every day. These data were synthesized from each of the included studies ( Biggerstaff & Thompson, 2008). From this synthesis, the researchers emphasized what it means to be an infertile man for those with gender phenomena as part of the experience. Therefore, we focused on the results of in-depth interviews conducted by each author in their study.

In this second procedure, the researchers/writers identified texts that had certain expressions or meanings (meaning units), which are mapped into a particular theme and categorized based on the findings (thematic coding). At this stage, the researchers also removed the phrases that are not relevant to the goals of this study. Finally, each finding that had the same expression was grouped and then presented in the form of tables or diagrams.

## 3. Results

### 3.1 Description of the study and quality

Based on the gender and masculinity theme, the researchers have tried to find evidence of the involvement of gender in cases of male infertility ( Table 1). For the first topic, masculinity and male infertility, 24 studies were analyzed. All studies that were included for analysis were qualitative studies originating from Asia, Europe, Africa, and America. Some of these studies performed in-depth interviews in person, but some studies did online interviews. The quality of qualitative studies using the CASP form is carried out in all eligible journals. Table 2 presents the summary results of our study assessment.

**Table 1.** Characteristics of eligible studies.

No.	Year	Author	Population	Sample size	Title	Results of investigation (include masculine/gender theme)
1	2011	Johansson, Hellström, and Berg	Sweden	8	Severe male infertility after failed ICSI treatment—A phenomenological study of men’s experiences.	Men undergoing ART programs feel inadequate as men, stuck with masculine stereotypes as part of fertility.
2	2013	Hinton and Miller	UK	38	Mapping of men’s anticipations and experiences in the reproductive realm: (In) fertility journeys.	This environment makes it challenging for males to consider the possibility of not being able to father a child.
3	2011	Shirani and Henwood	UK	53	Taking 1 day at a time: Temporal experiences in the context of unexpected life course transitions.	Men’s experiences as fathers have an impact on their future lives.
4	2004	Throsby and Gill	UK	13	“It’s different for men” Masculinity and IVF.	Male perception of infertility is heavily influenced by hegemonic masculine culture. Some men struggle with the perception that having children with a spouse is a necessary aspect of their role.
5	1994	Carmeli and Birenbaum-Carmeli	Israel and Canada	32	The predicament of masculinity: Toward understanding the male’s experience of infertility treatments.	The advent of female-centered infertility medicines has turned a male-inspired link of procreation with women into a disadvantage for men. From a feminist standpoint, these therapies were critiqued. At the same time, as this research demonstrates, the same medicines result in man’s isolation.
6	2001	Daniluk	Canada	65	“If we had it to do over again . . .”: Couples’ reflections on their experiences of infertility treatments.	Mixed feelings of uncertainty regarding the success of the therapy and the cost of treatment.
7	2010	Fahami *et al.*	Iran	10	Lived experience of infertile men with male infertility cause.	Men feel depressed and worthless. After finding that they are infertile, some men have sexual problems.
8	2004	Gannon, Glover, and Abel	UK	26	Masculinity, infertility, stigma, and media reports.	Men were portrayed as weak and powerless to circumstances beyond their control. The narratives used a variety of stereotypically masculine references, such as battle and mechanical parallels.
9	2005	Cudmore	UK	Not stated	Becoming parents in the context of loss.	Infertile men felt as if their masculinity had been questioned.
10	1999	Webb and Daniluk	Canada	6	The end of the line: Infertile men’s experiences of being unable to produce a child.	Inability to become a father is interpreted as a loss of manhood and authority. They call themselves losers, rubbish, and cowardly on some occasion. They also have issues with their sexual function as well.
11	2003	Herrera	Chile	49	“Men always adopt”: Infertility and reproduction from a male perspective.	Fatherhood is a central aspect of a Chilean man’s life and identity. However, they are still looking for ways to validate their position in the reproductive process.
12	2008	Malik and Coulson	Online/threads/messages	53 threads, 728 messages	The Male experience of infertility: A thematic analysis of an online infertility support group bulletin board.	The males felt uninvolved in the treatment, as if they were being ignored and treated as spectators.
13	2020	Hanna and Gough	UK	41	The social construction of male infertility: a qualitative questionnaire study of men with a male factor infertility diagnosis.	Men who are infertile feel as if they have failed to be masculine and invisible.
14	2013	Mumtaz, Shahid, and Levay	Pakistan	8	Understanding the impact of gendered roles on the experiences of infertility among men and women in Punjab.	Men’s infertility experience is largely determined by the gender roles men are encouraged to fulfill.
15	2016	Hanna and Gough	An online forum with 13 threads that were selected for analysis, totaling 415 posts, and there were 20 unique posters	13 threads: 415 posts, 20 unique posters	Emoting infertility online: A qualitative analysis of men’s forum posts.	Infertility deprives men of their pride, dignity, privacy, self-assurance, and future. This emotion is related to the tyranny of infertility.
16	2013	Tabong and Adongo	Ghana	45	Understanding the Social Meaning of Infertility and Childbearing: A Qualitative Study of the Perception of Childbearing and Childlessness in Northern Ghana.	Infertility is not just described as a couple’s inability to conceive children by the participants. But it also covers the failure to have male offspring or conform to society’s significant family expectations. The failure of men to conceive sons is blamed on them.
17	2020	Harlow *et al.*	Online from USA/Canada	14	A qualitative study of factors influencing male participation in fertility research.	Men expressed concerns about infertility as a result of their masculinity. It is challenging to discuss pregnancy and fertility with the men in this study.
18	2016	Arya and Dibb	UK	15	The experience of infertility treatment: the male perspective.	Social stigma has a big impact on how they think about infertility. Infertility is a symptom of men’s deterioration. This feeling affects their ability to communicate and express themselves.
19	2003	Inhorn	Egypt	2 infertile couples	“‘The Worms Are Weak’: Male Infertility and Patriarchal Paradoxes in Egypt.”	From the perception of a female spouse, men don’t always want to be blamed for infertility. There is a strong correlation between patriarchal culture and beliefs toward infertility treatment.
20	2013	Inhorn	Lebanon	120	“Why Me? Male Infertility and Responsibility in the Middle East.”	Men’s perspectives of infertility are influenced by their culture and beliefs. Some men are still hesitant to acknowledge that they are infertile, which may be affected by the negative stereotype of sterile males.
21	2004	Inhorn	Egypt, Lebanon	±18 couples with male infertility	“Middle Eastern Masculinities in the Age of New Reproductive Technologies: Male Infertility and Stigma in Egypt and Lebanon.”	Male infertility is complicated for Middle Eastern men, whereby fertility is traditionally associated with manhood. Infertility in men is thus a potentially emasculating condition that is shrouded in secrecy and stigma.
22	2009	Birenbaum-Carmeli and Inhorn	Israel, Lebanon	Not stated briefly	“Masculinity and Marginality: Palestinian Men’s Struggles with Infertility in Israel and Lebanon.”	Male infertility was considered a serious life disturbance by most of the males in this survey, both in Israel and Lebanon. Male infertility is associated with masculinity as a burden of secrecy.
23	2020	Baranwal and Chattopadhyay	India	150	Proposition of Belief and Practice Theory for Men Undergoing Infertility Treatment: a Hospital-based Study in Mumbai, India’	They are baffled and experience a range of feelings, including stress, separation, and frustration. Some of them also received unfavorable feedback from family members, prompting them to doubt their masculinity.
24	2017	Dolan *et al.*	UK	22	‘It’s like taking a bit of masculinity away from you’: toward a theoretical understanding of men’s experiences of infertility.	In the context of infertility, the male body can be regarded as a failing entity in and of itself (unable to father a child) as well as a subservient social entity (unable to measure up to hegemonic ideals) that defines men’s masculine identities.

**Table 2.** Methodological quality assessment (CASP) of included studies (n = 24).

Quality criterion	Assessment summary for study		
	Yes	No	Can’t tell
Were the aims of the research stated clearly?	24	0	0
Is a qualitative methodology appropriate?	23	0	1
Was the research design appropriate to address the aims of the research?	21	0	3
Did the recruitment strategy fit with the aims of the research?	20	0	4
Were the data collected in a way that addressed the research issue?	22	0	2
Has the relationship between the researcher and the participants been adequately considered?	16	0	8
Have the ethical issues been taken into consideration?	14	4	6
Was the data analysis sufficiently rigorous?	22	0	2
Were the findings elaborated clearly?	24	0	0
How valuable is this research?	All studies deliver a valuable contribution to knowledge and practice or policy		

### 3.2 Description of themes

From the results, 24 studies discussed gender issues in male infertility. All studies are in the form of qualitative studies conducted through in-depth and online interviews. The study population was from various continents. The study was also conducted on men who had followed treatment up to the stage of assisted reproductive technology (ART). The theme of masculinity is a prominent issue in these studies, regardless of the country. Many of the men who experience infertility question their manhood. The researchers try to summarize some of the themes that they found concerning masculinity in men with infertility. There are two themes that were identified based on our analysis, and then we explore these themes in seven descriptive themes ( Table 3).

**Table 3.** Selection of illustrative quotes for subthemes presented.

Analytic theme	Descriptive theme	Illustrative quote	List of study
Personal perception and beliefs	Being infertile is the same as not being a man anymore/crisis.	“Nobody just blamed me or told me anything, but I don’t think I’m even a guy, or others may think I’m not a man. I feel that I am a miserable person. I feel I’m so inept and unworthy, and people would think I can’t be on my own …” (p. 267). “A man should be able to have children … to give his wife children. So because I couldn’t I wasn’t a real man … that’s why I felt an attack on my maleness. ..t all comes down to one word … inadequate” (p. 15).	Cudmore (2005), Fahami *et al.* (2010), Hanna and Gough (2020), Hanna and Gough (2016) Gannon, Glover, and Abel (2004), Johansson, Hellström, and Berg (2011)
	Being a man is being a father (manhood is fatherhood) or impregnating a wife is a must for men.	“I’d say mentally it was certainly something you know that was taking its toll on me, something that was making me feel … you know when you can’t impregnate your wife, it makes me feel like less of a man. It makes me kind of feel like less of a person, and that’s something, which is core to your humanity … it’s something that’s kind of being a goal to your existence” (p. 244).	Webb and Daniluk (1999), Shirani and Henwood (2011), Hinton and Miller (2013), Herrera (2013), Dolan *et al.* (2017), Harlow *et al.* (2020), Hanna and Gough (2020)
Social and cultural norms, values, and expectations	Having children is a man mandate from society.	“The reason I wouldn’t want to tell someone was this weird stigma in our culture that, if a man doesn’t produce sperm, he’s less of a man . ..ou never want to be less of a man … . I didn’t want them to think less of me for not being able to produce sperm” (p. 244).	Throsby and Gill (2004), Inhorn (2003)
	Being infertile will be a stigma (judgment by society).	“No, he didn’t talk about it anything like as much as me, and he wouldn’t say, you know. He wouldn’t tell people about the cause. He’d just let people’s assumptions go. .. think he just couldn’t bear to talk about it” (p. 337).	Inhorn (2003), Inhorn (2004), Mumtaz, Shahid, and Levay (2013), Arya and Dibb (2016), Baranwal and Chattopadhyay (2020), Birenbaum-Carmeli and Inhorn (2009), Inhorn (2013), Tabong and Adongo (2013), Dolan *et al.* (2017)
	Failure to have children is resistance to God’s command.	“The bible says in genesis that we should multiply and fill the earth to ensure the continued existence of the earth” (p. 4).	Tabong and Adongo (2013)
	Between infertile man and feminism.	“I think there’s a perception—I think many people would agree with this—that infertility is a woman’s problem … . I think in terms of getting more men to participate, if you can quantify when it is a male problem, if you can make a stronger case there and then the wife or the partner can then use that as a way to persuade their husband or spouse hopefully” (p. 10).	Malik and Coulson (2008), Harlow *et al.* (2020), Carmeli and Birenbaum-Carmeli (1994)

### 3.3 Metasynthesis of study findings

The concept of gender phenomena in infertility is a topic that can cause friction. In generating offspring, collaboration between women and men is required. Infertility may be caused by one or both parties. Infertility is often associated with women’s bodies, so if the cause is men, there is often excessive gender conflict either from the man himself or even from his environment.

From the core analysis of all eligible studies, two major themes surround the “gender issue” central phenomenon in infertility cases. The two big themes are:

1. Personal perceptions and beliefs
2. Sociocultural norms, values, and expectations

These two main themes are phenomena experienced by infertile men related to masculinity. The researchers/writers synthesize the men’s experiences again on these two major themes. These themes were analyzed in describing the experiences that men felt about being infertile. The researchers/writers summarize the results and the studies involved in describing this phenomenon ( Table 3).

## 4. Discussion

We are faced with a situation where infertility is stigmatized and it is challenging to identify cases of it because of this ( Hinton & Miller, 2013). This phenomenon has not changed much in recent decades. Some communities still label infertility as a failure that makes men reluctant to be treated. Sometimes, men who struggle with infertility are often excluded from the community and considered as a “second sex” ( Rimer, 2010).

Based on the data synthesis in this study, two significant themes underlie gender issues in infertility cases. But both influence each other so that it harms men’s acceptance of themselves, especially in handling infertility cases. Some factors come from the man himself, and some influences are from society. The findings of this study indicate that the existence of social norms such as masculinity and stigma have an impact on men’s perceptions of infertility. Many respondents from the research revealed that infertility makes them feel they are not men. This is because the patriarchal system is still very prominent across the globe. Likewise, this binary gender and sex system is still dominantly accepted throughout the world. There is a culture that thinks hegemonic masculinity is considered as an appropriate construction to define men. It is claimed that any shift in value from this construction is considered a decrease in masculinity. These demands are not only obtained by men from the norms or surrounding culture but can also come from their understanding of religion or belief ( Arya & Dibb, 2016; Tabong & Adongo, 2013). Even some men experience their condition being underestimated by their wives ( Gannon *et al.*, 2004). This condition is what the researchers/writers consider to be traditional masculinities.

Traditional masculinities have a major negative impact on men’s willingness to share their situation with others. Because they do not want to be perceived as weak or less masculine, men are reluctant to consult with and seek assistance. The infertile men also face a dilemma when seeking help. Although Assisted Reproduction Technology is often provided to assist men, it actually becomes a burden for them. Apart from financial concerns, strong religious values also often put them in a difficult position ( Birenbaum-Carmeli & Inhorn, 2009). Even though the demands are still targeted at them, a man has his own value when he has biological children. In some countries, being a man automatically means one becomes a father. For example, in the Middle East, fatherhood is traditionally considered the equivalent of manhood. Men are viewed as powerful figures when they participate in reproduction. So, men will feel threatened when they are unable to reproduce ( Inhorn, 2004).

This can lead them to severe anxiety and depression ( Bahar *et al.*, 2019). This is the dangerous impact of traditional masculinities on infertile men, a condition that can eventually be harmful to the man.

Yang *et al.* conducted a study in China reporting the uncertain prevalence of infertile men who experienced depression. Depression (20.8%), anxiety (7.8%), and a combination of the two (15.5$) were present in men, respectively ( Yang *et al.*, 2017). Our findings also suggest that infertile men are significantly at risk for depression and anxiety disorders. The disorder may be brought on by the demands of gender itself or by other factors.

There are no definite reports on whether this gender role is needed to have the same impact on men. Some assumed an association between gender role expectation and mental disorders in men ( Ahmadi *et al.*, 2011; Folkvord *et al.*, 2005). In addition, one of the main factors contributing to males developing mental disorders is the costs they incur for treatment ( Bai *et al.*, 2019).

Men with fertility disturbances are often labeled as men with a “lack of sexuality.” They are considered not to have good sexual performance ( Pattnaik *et al.*, 2016). Although they differ, there is a connection between male infertility and sexual function. It is not uncommon for male infertility to cause a sexual dysfunction experience. Measurements using the International Index of Erectile Function score showed a correlation between infertility and the incidence of erectile dysfunction ( Coward *et al.*, 2019; Gao *et al.*, 2013; Ozkan *et al.*, 2015; Sahin *et al.*, 2017). Patients with primary male infertility are more likely to have symptoms of erectile dysfunction than secondary male infertility ( Sahin *et al.*, 2017). Various medical procedures undertaken during the pregnancy program itself can also alter sexual behavior and the couple intimacy, often leading to temporary sexual dysfunction ( Bechoua *et al.*, 2016). Additionally, sexual satisfaction can also be reduced due to the forced sexual activity for conception purposes ( Tao *et al.*, 2011).

**Figure 2.**
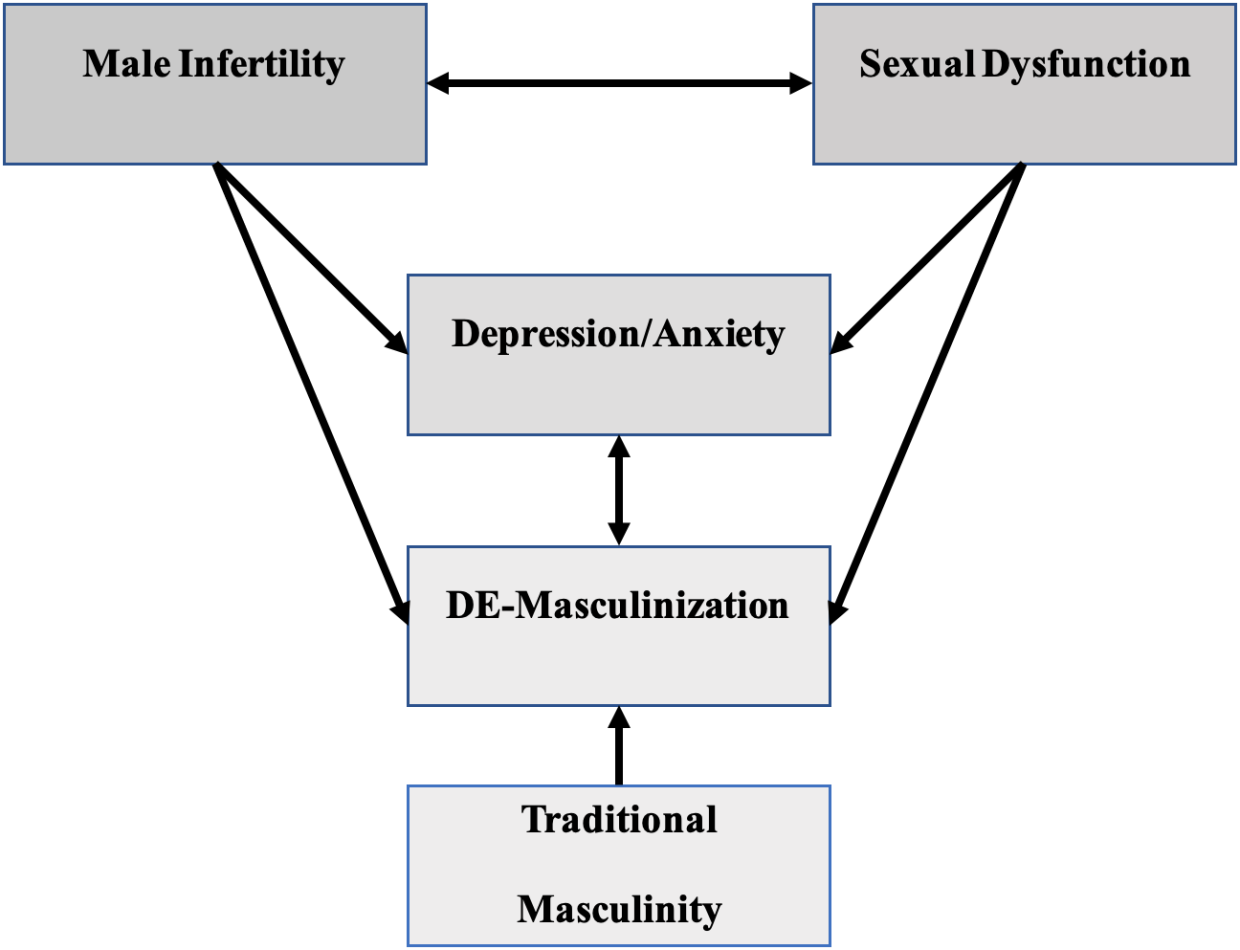
Association between male infertility, sexual dysfunction, mental health, and masculinity. Male infertility causes men to be stigmatized as failed men because of the masculinity demands. On the other hand, infertile men are also sometimes caused by sexual dysfunction which is often identified as men's lack of masculinity. These two conditions affect their mentality. Men with mental disorders, especially depression and anxiety, also get the label lack of masculinity.

Ejaculatory disorders have also been reported in several studies, aside from erectile dysfunction ( Gao *et al.*, 2013). This disorder can also be a part of the anxiety experienced by infertile men. This variable is intriguing because it can lead to infertility due to sexual function disorders ( Yang *et al.*, 2017), but this complaint may arise after being diagnosed with infertility. However, the two can be directly or indirectly related. In the community, impaired sexual function is often also considered a sign of DE-masculinization ( Pakpahan *et al.*, 2021). There is a very complex and interconnected relationship between male infertility, sexual dysfunction, depression, and anxiety ( Figure 2).

The World Health Organization states that our sociocultural circumstances still often demand that men do not get sick, especially those with mental illness ( Gough & Novikova, 2020). This is what makes men often hide their real condition. Problems will be complicated when they are infertile and depressed. Infertile men with depression and anxiety face a double burden of their condition. Their emotions and mentality as men are also questioned in the community ( Gourounti *et al.*, 2010).

Male infertility is not a condition that threatens men’s life, but because of the stigma and social punishment that come with it, men are put under a great pressure. This is especially in a patriarchal culture with social and religious values that are not easy to control. These two things are values that have been passed down from generation to generation and are firmly attached to the community. These values are like big strongholds in the community that are not easily torn down. It seems that there is a strong association between the “masculinity issue” and the way men face infertility.

### 4.1 Future directions

There is a phenomenon in a society that a person’s masculinity will affect health behavior. This phenomenon is not always true. Rochelle reports that norms of masculinity do not always go hand in hand with a person’s healthy behavior. Instead, Rochelle claims that a person’s support system influences a person’s healthy behavior ( Rochelle, 2020). In the case of male infertility, couples’ therapy and family education are very important to change the perspective of men who are infertile. If the patient’s support system is warm, feelings of DE-masculinization will not occur.

Changing perceptions and social constructions is not easy but requires attention. It is important to involve community organizations and religious leaders in creating the emotional dangers of social construction. Traditional masculinities put men as the victims. Men have the right to be independent of their conditions without being judged by the norms and values that isolate them. Men confiding in fellow men about infertility is beneficial. or others. Almost certainly fellow men better understand the conditions and emotions of each other. Men with infertility need a place to share and exchange ideas about their problems such as support groups.

The media has played a big role in changing any culture in this century. The “MeToo movement,” a massive American movement against sexual harassment, has hugely impacted twenty-first-century culture. Against “traditional masculinities campaign” should also need a platform and place like this to elevate men’s self-confidence. Men have the right to speak for themselves if it does not destroy others. According to Knudsen and Andersen, one of the genres where values of masculinity are particularly obvious and can be represented is advertising or campaigns that are employed as vehicles of cultural myth. The movement against traditional masculinities requires the media to convey the detrimental impact of this phenomenon. Lynx’s *Is It Ok for Guys* (2017) and Gillette’s *The Best a Man Can Be* (2019) are two campaigns that have asserted no to overestimating masculinity with different audiences. Although it has received negative criticism, consistent ideas like these need to be communicated ( Knudsen & Andersen, 2020). In 2021, Jane Campion’s *The Power of the Dog* was also appreciated for trying to dispel the toxicity of excessive masculinity. We need movie ideas that depict dangerous extreme masculinity, especially in the case of infertility (so far, not many films have told). Cervi and Knights argue that a combination of social, medical, and organizational perspectives that are heterogeneous and engaged in reproduction is needed for the treatment of infertility. All of them can play a role in providing medication and nutrition, providing legal advice or moral therapy to relieve stress ( Cervi & Knights, 2022). Thus, this solution is required to fight the toxicity of excessive masculinity.

There are many possibilities of finding contradictions between feminism and counter-traditional masculinity in couples who are dealing with infertility, especially if the male causes the infertility. One very interesting fact related to infertility is that although the main cause is men, the focus is women. Even women bear the severe consequences of infertility treatment, for example, ART (Assisted Reproduction Technology) ( Hanna & Gough, 2020). When couples must choose ART, at this stage, women are required to undergo hormonal stimulation that they should not experience. Hormonal stimulation is known to have very agonizing effects for a woman’s body, and it can even be life-threatening ( Mahajan, 2013). Because of the failure of male reproductive function, women must bear the consequences. Sometimes, fertile women take this path to maintain monogamous marriages and obtain offspring ( Lorber, 1989).

One of the interesting proposals from Hanna and Gough (2015) is to involve men more openly in reproductive research. This will have practical implications for service delivery. It is necessary to provide men a voice and support them to connect the needs of men and the management of infertility by men ( Hanna & Gough, 2015).

Above all, we need to understand that the movement against traditional masculinity is beneficial for all of society. Traditional masculinity is a very dangerous condition not only for men but also for women and children. Traditional masculinity is actually an over-glorification of men that causes negative effects on others ( Sculos, 2017).

This study is still limited to synthesizing the experience of phenomena that individuals feel about infertile men. But a more profound grounded theory of why infertility must be synchronized with masculinity is still needed. In addition, issues close to society and culture are still limited to a few populations and not globally, so the generalization of masculinity with infertility is still not evenly distributed. Similar studies with varied individuals and cultures still need to be conducted again for a global masculine phenomenon. Moreover, some of the studies that we include in this review do not explicitly explain the number of samples included and the process of obtaining the data.

## 5. Conclusion

Infertility is a common condition. Modernization and public awareness have encouraged the knowledge of this condition, although there are still hidden cases is often caused by cultural and social views that infertility is humiliation, especially if the male is the main problem. In a patriarchal culture, male infertility cause is considered a failure in men. This is frequently a result of society’s overly high expectations for men. This unwarranted expectation arises because of the over-glorification of men in the society, even though men are the same as women who both have limitations.

Traditional masculinities pose a huge threat to men. The feeling of “isolation, loneliness, powerlessness, and recklessness” even depression and other psychological disorders are real impacts for men with infertility. We must realize that against traditional masculinity does not mean threatening the feminist movement. There is nothing to do to fight traditional masculinity toward feminism. Instead, against traditional masculinity helps feminism find its place.

## Data Availability

Figshare: PRISMA_2020_checklist Masculinity.docx. https://doi.org/10.6084/m9.figshare.22193602.v1 ( Pakpahan, 2023). This project contains the following extended data:
‐
Figure 1 Prisma.doc PRISMA checklist for “Am I Masculine?” A metasynthesis of qualitative studies on traditional masculinity on infertility. https://doi.org/10.6084/m9.figshare.22193602.v1 ( Pakpahan, 2023). Data are available under the terms of the Creative Commons Attribution 4.0 International license (CC-BY 4.0).
