# Supplementary material for: “Am I Masculine?” A metasynthesis of qualitative studies on traditional masculinity on infertility
Source: F1000Res. 2023 Mar 9;12:252. [Version 1] doi: 10.12688/f1000research.131599.1 (PMC10050908; doi:10.12688/f1000research.131599.1)
Supplement: Peer review history

# Peer review: Reviewer response for version 1

**DOI:** 10.5256/f1000research.144456.r166016

**Published:** 2023-03-28

**Authors:** Suriyani Muhamad

**Affiliations:** 1 Faculty of Business, Economics and Social Development, Universiti Malaysia Terengganu, Kuala Terengganu, Malaysia

1. I can say that this review paper offered a different perspective on the male infertility issue by integrating the masculinity aspect.
2. It is a great review paper that adds to the present review papers on male infertility studies.
3. I suggest elaborating more on the below paragraph, perhaps with an example: 
  "Above all, we need to understand that the movement against traditional masculinity is beneficial for all of society. Traditional masculinity is a very dangerous condition not only for men but also for women and children. Traditional masculinity is actually an over-glorification of men that causes negative effects on others ( Sculos, 2017)."
4. Overall, this review paper is recommended to be indexed.

Are the rationale for, and objectives of, the Systematic Review clearly stated?

Yes

Is the statistical analysis and its interpretation appropriate?

Not applicable

Are sufficient details of the methods and analysis provided to allow replication by others?

Yes

Are the conclusions drawn adequately supported by the results presented in the review?

Partly

Reviewer Expertise:

overall, it can be understood, except for medical and specific terms of medical

I confirm that I have read this submission and believe that I have an appropriate level of expertise to confirm that it is of an acceptable scientific standard.

# Peer review: Reviewer response for version 1

**DOI:** 10.5256/f1000research.144456.r166017

**Published:** 2023-03-28

**Authors:** May Soe Aung, Swe Mar Myint Lwin

**Affiliations:** 1 Department of Preventive and Social Medicine, University of Medicine, Yangon, Myanmar; 2 Department of Health Behaviour and Communication, University of Public Health, Yangon, Myanmar

The article addresses the linkage of masculinity, infertility and mental health consequences by means of metasynthesis based on qualitative studies. The authors can explore the important points in the abstract. Methodology is sound and discussion is comprehensive including good description about limitations based on the study findings. Some issues that need to be modified are as follows:

**Introduction:**

1. Mental health problems like anxiety and depression should be added because these are highlighted in the discussion.
2. Although the detail of the masculinity and infertility are well introduced, more justifiable points with reasons linking to the aim of the study are recommended.
3. Repetition of third sentence from the first paragraph in the second paragraph should be modified.
4. In section 1.1, fifth sentence by Moynihan is suggested to be rewritten to avoid direct copy of the original format.

**Flow Diagram: **

1. It is needed to check and confirm why the number of studies after duplicates removed (n=145) is lower than that after screening (n=242).

**Methods:**

1. It is suggested to mention the search tool used to identify the components of the research question such as SPIDER (Cooke *et al.* 2012 ^1^).
2. It is better to mention whether the authors searched for gray literatures and doctoral dissertations.
3. Range of years should be mentioned in the inclusion criteria of the studies.
4. For enhancing transparency in reporting the synthesis of qualitative research, the ENTREO statement is recommended. However, the authors can provide the justification for preferring PRISMA checklist.

**Results:**

For Table 1:

1. It will be more suitable to order the included studies according to the year of study.
2. Color using for author name should be consistent; because that of no. 6 is different from others.
3. Under Sample Size in no.12 and 15, threads, messages, posts, unique posters are suggested to clarify for understanding of readers because the data collection method mentioned in the inclusion criteria is in-depth interview.
4. It will be better to modify as “Study area” in place of “Population”.
5. In the last column, mentioning “Results of investigation” is enough and so explanation within parentheses are suggested to remove.

**Discussion: **In the fourth paragraph, first line should be written with description instead of “This” at the start.

**Conclusion: **The contents of the conclusion in the abstract and the body of manuscript should be consistent.

Are the rationale for, and objectives of, the Systematic Review clearly stated?

Yes

Is the statistical analysis and its interpretation appropriate?

Not applicable

Are sufficient details of the methods and analysis provided to allow replication by others?

Yes

Are the conclusions drawn adequately supported by the results presented in the review?

Yes

Reviewer Expertise:

Maternal Health , Reproductive Health,  Infection control measures, One Health Approach

We confirm that we have read this submission and believe that we have an appropriate level of expertise to confirm that it is of an acceptable scientific standard, however we have significant reservations, as outlined above.

## References

[1] : Beyond PICO: the SPIDER tool for qualitative evidence synthesis. *Qual Health Res* .2012;22(10) : 10.1177/1049732312452938 1435-43 10.1177/1049732312452938 22829486

# Peer review: Reviewer response for version 1

**DOI:** 10.5256/f1000research.144456.r166012

**Published:** 2023-03-20

**Authors:** Abiodun Mathias Emokpae

**Affiliations:** 1 Department of Medical Laboratory Science, School of Basic Medical Sciences, University of Benin, Benin City, Edo, Nigeria

This study examines the effect of traditional masculinity on male infertility. This perception varied in size and shape from one society to another, but all geared towards discriminating against the infertile man. The article is well written and has contributed to knowledge in male infertility study. However, the keywords should be derived using “MeSH on Demand”.

 The association of male infertility with masculinity is inappropriate and it makes infertile men regard themselves as inadequate, incomplete and less masculine. The disorder therefore, poses danger to masculinity because of wrong perception and/or belief. The perception of inability to achieve pregnancy as failure of manhood is misleading and has contributed to several associated psychological and adverse health effects. Infertility can be due to male or female gender, therefore requires cooperation and patience by the couples. It should not be stigmatized and affected men should be encouraged to present themselves for evaluation and treatment. Open discussion against traditional masculinity may be beneficial to solving several psychological problems associated with male infertility.

Are the rationale for, and objectives of, the Systematic Review clearly stated?

Yes

Is the statistical analysis and its interpretation appropriate?

Yes

Are sufficient details of the methods and analysis provided to allow replication by others?

Yes

Are the conclusions drawn adequately supported by the results presented in the review?

Yes

Reviewer Expertise:

Reproductive Biology

I confirm that I have read this submission and believe that I have an appropriate level of expertise to confirm that it is of an acceptable scientific standard.
